# Response to Comment on “Dying in the Sun: Direct evidence for elevated UV-B radiation at the end-Permian mass extinction”

**DOI:** 10.1126/sciadv.adj6309

**Published:** 2023-08-25

**Authors:** Phillip E. Jardine, Huiping Peng, John E. A. Marshall, Barry H. Lomax, Benjamin Bomfleur, Matthew S. Kent, Wesley T. Fraser, Feng Liu

**Affiliations:** ^1^ Palaeobotany Group, Institute of Geology and Palaeontology, University of Münster, Münster 48149, Germany.; ^2^ Nanjing Institute of Geology and Palaeontology, Chinese Academy of Sciences, Nanjing 210008, China.; ^3^ School of Ocean and Earth Science, University of Southampton, National Oceanography Centre, Southampton SO14 3ZH, UK.; ^4^ Division of Agricultural and Environmental Sciences, School of Biosciences, The University of Nottingham, Sutton Bonington Campus, Sutton Bonington, Leicestershire LE12 5RD, UK.; ^5^ Geography, School of Social Sciences, Oxford Brookes University, Oxford OX3 0BP, UK.; ^6^ State Key Laboratory of Palaeobiology and Stratigraphy and Center for Excellence in Life and Paleoenvironment, Nanjing 210008, China.

## Abstract

Seddon and Zimmermann have raised questions about the evidence for increased UV-B flux across the end-Permian mass extinction (EPME) that was presented in our recent study, specifically regarding the measurement of UV-B–absorbing compound (UAC) levels in fossil pollen. We respond to these points, arguing that the comparison of FTIR spectra of >250 million–year–old Permian fossil pollen with ~700-year-old subfossil pollen is not valid and that negligible nonrandom interference derived from water vapor fluctuations during data generation cannot coincidentally produce a substantial UAC peak during the EPME. Furthermore, we refute the suggestion that the measured aromatic peak at 1600 cm^−1^ could have been influenced by diagenetic products from other organic constituents of pollen. The most productive route forward will be to generate sporomorph geochemical data from additional Permian-Triassic boundary sections to test the results put forward in our study.

## Teaser

Data artefacts and diagenesis do not explain an increase in UV-B absorbing compounds during the end-Permian mass extinction.

Seddon and Zimmermann (*1*) call into question our recently published results (*2*) that provide evidence for increases in ultraviolet-B (UV-B) flux across the end-Permian mass extinction (EPME). Specifically, they question whether the aromatic peak present in Fourier transform infrared (FTIR) spectra at 1510 to 1520 cm^−1^ (referred to in our paper and here as the 1510-cm^−1^ peak), which is commonly used to measure UV-B–absorbing compound (UAC) levels in pollen and spores (sporomorphs) (*3*–*6*), was present and measurable in our spectra and whether the larger peak at 1590 to 1600 cm^−1^ (hereafter the 1600-cm^−1^ peak) represents aromatic compounds in sporopollenin. Here, we respond to each of these points in turn.

Seddon and Zimmermann suggest that the 1510-cm^−1^ peak that we measured in our spectra is indistinguishable from nonrandom interference from water vapor. They came to this conclusion by comparing FTIR spectra of Permian-Triassic fossil pollen in our paper (note that these are pollen grains, not spores, as Seddon and Zimmermann incorrectly state in their comment) with those of ~700-year-old subfossil *Pinus* pollen (*1*, *7*). Although the fossil pollen (*Alisporites tenuicorpus* Balme 1970) measured in our paper has potential botanical affinities with ancestral conifers (*2*), we do not believe that the FTIR spectra of these two types of pollen can be directly compared because of the vast age gap (over 250 million years) between them. It is unrealistic to expect that the FTIR spectra of fossilized pollen from the latest Permian would have the same spectral properties as subfossil pollen grains that have not undergone diagenesis or repolymerization. Seddon and Zimmermann also argue that that there is no peak present in our spectra at 1510 cm^−1^ but that there is one at ~1515 cm^−1^; however, as noted above, the target UAC peak occurs within a broader range of wave numbers (typically 1510 to 1520 cm^−1^ and often in the 1512- to 1515-cm^−1^ region), and we referred to in this way for convenience and to maintain consistency with previous papers (*4*–*6*). Furthermore, while variation in atmospheric water vapor during data collection would be expected to add random noise to the peak height measurements, Seddon and Zimmermann provide no explanation for how this would systematically bias the measurements upward or downward at any point in the studied section (or, specifically, across the EPME interval). We also note that the moderate positive correlation (Pearson’s *r* = 0.44) between the 1725- and 1510-cm^−1^ peaks in second derivative spectra does not imply a high correlation between peak heights in nonderivated spectra, which is what was measured in our paper (*2*).

Seddon and Zimmermann acknowledge that the 1600-cm^−1^ peak in our spectra represents aromatic compounds but suggest that it could have been influenced by the “diagenetic products from other organic constituents of pollen (including plant waxes and lipids), as well as products linked to thermal maturation” (*1*). There are several weaknesses in this line of argument. First, Seddon and Zimmermann do not explain how aliphatic compounds such as lipids will contribute to the measured aromatic peak at 1600 cm^−1^. Second, pollen-bound lipids are labile compounds that, along with the proteins and carbohydrates found in fresh pollen grains, are quickly lost in sedimentary records, and it is far from certain that they are involved in sporopollenin repolymerization (*8*–*10*); it is also not clear what Seddon and Zimmermann mean by “plant waxes” as constituents of pollen (if not lipids). Third, it is not clear why the concentrations of these pollen-derived aliphatic compounds would change in the EPME interval, and because thermal maturity levels do not change through the Qubu section (*2*), there is no reason why different thermal maturation conditions would only affect the analyzed pollen grains during the EPME. Fourth, even allowing for the incorporation of external organic material into sporopollenin during repolymerization, the increase in the height of this peak at the Permian-Triassic boundary (PTB) still demands an explanation: Why do we see a substantial increase in aromatics at this point in time?

Seddon and Zimmermann’s argument relies on a change in atmospheric water vapor during data generation, coincident with shifting spectral baselines (although not so consistent across the spectra that the dynamics of the 1510- and 1600-cm^−1^ aromatic peaks cannot be decoupled in specific parts of the section, e.g., at ~40 m). This is considerably less parsimonious than the explanation put forward in our paper, where the increase in aromatic content at the PTB, recorded from different peaks and measurement windows, is tied to a specific scenario of an increase in UACs driven by enhanced UV-B flux at this time.

Seddon and Zimmermann close by cautioning that others should avoid the methods used in our paper but fail to suggest any alternatives, either in terms of how the data should be generated or those data processed and analyzed. Seddon and Zimmermann offer a restrictive set of conditions for generating UAC data from sporomorphs, which essentially limits such analyses to subfossil and extant material [even in the case of the exceptionally preserved Carboniferous megaspores (*11*) highlighted by Seddon and Zimmermann, we note that the 1510-cm^−1^ UAC peak was not detected in nonderivated spectra, and we are unaware of any FTIR spectra from pre-Quaternary fossil sporomorphs that resemble the spectrum of the 700-year-old pollen grains shown by Seddon and Zimmermann [figure 1 of (*1*)]. Rather than excluding the quantitative analysis of fossil sporomorph data a priori, we advocate for a more open and inquiry-driven approach, including generating data from additional PTB sections to compare with, and validate the results from our study, and extending these analyses to other proposed UV-B perturbations such as the end-Devonian mass extinction (*12*). We also note that, at present, micro-FTIR provides the only practical means of generating UAC datasets from fossil specimens, given the large sample sizes needed for gas chromatography–mass spectrometry–based analyses (*13*) and the challenges with applying Raman spectroscopy to isolated sporopollenin and related bio/geopolymers, because of strong autofluorescence (*14*, *15*). Naturally, we encourage the development of other approaches for generating UAC data from FTIR spectra, but to date, the peak height–based approach used in our paper is the only one that has been developed and tested on a range of extant, subfossil, and fossil material (*2*–*6*).
